# Heat-Killed *Lactobacillus delbrueckii* subsp. *lactis* 557 Extracts Protect Chondrocytes from Osteoarthritis Damage by Reducing Inflammation: An In Vitro Study

**DOI:** 10.3390/nu16244417

**Published:** 2024-12-23

**Authors:** Yu-Chen Hu, Tzu-Ching Huang, Bau-Shan Hsieh, Li-Wen Huang, Jin-Seng Lin, Han-Yin Hsu, Chia-Chia Lee, Kee-Lung Chang

**Affiliations:** 1 Department of Biochemistry, School of Medicine, College of Medicine, Kaohsiung Medical University, Kaohsiung 807378, Taiwan; huyujena@gmail.com (Y.-C.H.); huangtavia@gmail.com (T.-C.H.); hsiehbs@gmail.com (B.-S.H.); 2 Graduate Institute of Medicine, College of Medicine, Kaohsiung Medical University, Kaohsiung 807378, Taiwan; 3 Department of Medical Laboratory Science and Biotechnology, College of Health Sciences, Kaohsiung Medical University, Kaohsiung 807378, Taiwan; lewehu@cc.kmu.edu.tw; 4 Culture Collection & Research Institute, Synbio Tech Inc., Kaohsiung 821011, Taiwan; jslin@synbiotech.com.tw (J.-S.L.); hanin@synbiotech.com.tw (H.-Y.H.)

**Keywords:** osteoarthritis, *Lactobacillus*, chondrocyte, anti-inflammation

## Abstract

Background: Osteoarthritis (OA) is a chronic condition characterized by joint pain and disability, driven by excessive oxidative stress and inflammatory cytokine production in chondrocytes, resulting in cell death and cartilage matrix breakdown. Our previous study showed that in monosodium iodoacetate (MIA)-induced OA rats, oral administration of heat-killed *Lactobacillus delbrueckii* subsp. *lactis* 557 (LDL557) could significantly decrease OA progression. Methods: Accordingly, we designed an in vitro cell culture study aimed at investigating the effects of heat-killed LDL557 extracts on chondrocytes using SW1353 cells (a human chondrosarcoma cell line) challenged with 5 μM MIA to mimic OA conditions. Results: The results showed that the 10 μg/mL LDL557 extracts protected SW1353 cells from MIA-induced death and reduced extracellular matrix (ECM) loss, as evaluated by toluidine blue O staining and extracellular matrix component synthesis with RT-qPCR measurement. This was achieved by decreasing the expression of MIA-induced pro-inflammatory cytokines, including IL-1β, IL-6, and TNF-α, while slightly increasing the MIA-suppressed expression of the anti-inflammatory cytokine IL-10, which were evidenced by RT-qPCR analysis. Moreover, the RT-qPCR evaluation also indicated that the LDL557 extracts slightly reduced the expression of COX-2 compared with the control, while it did not reduce the MIA-increased expression of microsomal prostaglandin E synthase-1 (mPGES-1). In addition, the LDL557 extracts influenced neither the matrix-degrading protease expressions measured via RT-qPCR nor the oxidative stress measured via fluorescence flow cytometry in the cells with or without the MIA challenge. Conclusions: This study demonstrates that LDL557 extracts may protect chondrocytes from OA damage by reducing inflammation-related factors and thus mitigating cartilage matrix loss, suggesting LDL557 extracts are attractive alternatives for OA applications.

## 1. Introduction

Osteoarthritis (OA), a degenerative joint disease of the knee, is the most common form of arthritis in aging adults. It is identified by the degradation and progressive loss of articular cartilage, leading to persistent pain and reduced mobility. In 2020, approximately 595 million people worldwide suffered from OA, equivalent to 7.6% of the global population, and this number is expected to increase by 74.9% in 2050 [1]. In healthy articular cartilage, over 90% of the volume is extracellular matrix (ECM), primarily composed of collagen type II and aggrecan, while chondrocytes account for 5%. SOX9, a key transcription factor, promotes collagen type II expression and cartilage formation [2,3]. A report by Cucchiarini et al. showed that transferring the human SOX9 gene into human chondrocytes stimulated the production of collagen type II and proteoglycan [4].

In early OA, chondrocytes secrete tissue inhibitors of metalloproteinases (TIMPs) to regulate matrix metalloproteinases (MMPs) and certain members of a disintegrin and metalloproteinase (ADAM) with thrombospondin motifs (ADAMTSs) while promoting proteoglycan synthesis to repair cartilage [5,6]. However, excessive MMPs and ADAMTSs disrupt the ECM balance, degrading collagen and aggrecan and accelerating OA progression [7,8]. Inflammatory mediators like IL-1β, IL-6, and TNF-α also increase MMP and ADAMTS expression, leading to cartilage breakdown and worsening joint symptoms [9,10]. IL-1β can also induce chondrocytes to release cyclooxygenase-2 (COX-2) and inducible nitric oxide synthase (iNOS), increasing prostaglandin E2 (PGE2) and nitric oxide (NO) [11]. PGE2, formed from arachidonic acid by COX-2 and microsomal prostaglandin E synthase-1 (mPGES-1), promotes inflammation and cartilage breakdown in OA [12]. Conversely, IL-10 has anti-inflammatory and chondroprotective effects [13], enhancing collagen type II and aggrecan synthesis, reducing MMP activity, and preventing chondrocyte apoptosis, offering potential therapeutic benefits for OA [14]. In addition, the excess reactive oxygen species (ROS), such as superoxide anion (O_2_^−^), hydroxyl free radical (HO^−^), hydrogen peroxide (H_2_O_2_), or nitric oxide (NO), may drive cartilage degradation, which is commonly found in OA [15].

It is known that high bacterial diversity in the gut supports digestion, nutrient absorption, metabolic regulation, and immune function while preventing infections [16]. Conversely, dysbiosis—an imbalance in gut microbiota—has been linked to diseases like inflammatory bowel disease, obesity, type 2 diabetes, cardiovascular diseases, and certain cancers [16]. Emerging research underscores the connection between gut microbiota and OA development, introducing the “gut-joint axis” concept, which highlights how gut bacteria influence immune responses, inflammation, and joint health [17]. Dysbiosis, or an imbalance in gut microbiota, can exacerbate inflammation through pro-inflammatory cytokines like TNF-α, IL-1, and IL-6, contributing to OA progression [18]. Regulating gut microbiota through probiotics, prebiotics, dietary changes, and other interventions offers potential therapeutic benefits [17]. Both preclinical and clinical studies indicate that probiotics could alleviate OA-related pain [17]. For instance, live *Lactobacillus acidophilus* LA-1 significantly reduced joint pain in an MIA-induced OA rat model by desensitizing nociception and lowering the levels of pain modulators and pro-inflammatory factors [19]. However, their sensitivity to heat and oxygen challenges their stability [20]. Postbiotics, derived from inanimate probiotics, offer a stable alternative with similar benefits, including antibacterial, antioxidant, and immunomodulatory effects, supporting gut health, metabolism, and immunity with a longer shelf life and easier maintenance [21]. Moreover, heat-killed *Lacticaseibacillus paracasei* MCC1849 was reported to alleviate subjective cold symptoms in healthy adults, showcasing its immunomodulating effects [22]. Similarly, lyophilized, heat-killed *Lactobacillus acidophilus* LB combined with its culture medium shortened the healing time in infants with non-rotavirus diarrhea by one day [23,24]. In children under 5 years old, heat-killed *Lactobacillus acidophilus* LB was found to therapeutically reduce diarrhea duration, while heat-inactivated *Lacticaseibacillus paracasei* CBA L74 was effective in preventing diarrhea, pharyngitis, and laryngitis [25,26]. In our prior research, heat-killed LDL557 demonstrated efficacy in reducing knee joint swelling, protecting chondrocytes, slowing cartilage degradation, and mitigating OA progression in a rat model of MIA-induced OA [27]. Building on these promising results, this study aims to explore further the effects and underlying mechanisms of heat-killed LDL557 on chondrocytes.

The World Health Organization (WHO) designated 2021–2030 as the “Decade of Healthy Aging”, aiming to prioritize life expectancy and improving life quality, particularly for aging populations. Addressing age-related diseases like OA is crucial to this mission, as OA significantly impairs mobility and daily functioning, reducing quality of life. *Lactobacillus delbrueckii* subsp. *lactis* 557 (LDL557) is a biosafety level 1 probiotic strain approved by the American Type Culture Collection (ATCC) and meets the European Union’s Qualified Presumption of Safety (QPS) standards for food and feed [28]. It has also been approved as a food ingredient in Taiwan, making it a promising candidate for addressing OA in aging adults. Herein, we explore the immunobiotic potential of heat-killed LDL557 extracts (hereafter referred to as LDL557 extracts) to affect inflammation, oxidative stress, and other related protective mechanisms in chondrocytes, using a monosodium iodoacetate (MIA)-induced OA model in the human chondrocyte cell line (SW1353 cells).

## 2. Materials and Methods

### 2.1. Preparation of Heat-Killed Lactobacillus Extract

Heat-killed *Lactobacillus delbrueckii* subsp. *lactis* 557 (LDL557), obtained from SYNBIO TECH INC. (Kaohsiung, Taiwan), was resuspended in phosphate-buffered saline (PBS) at a concentration of 20 mg/mL. The suspension was homogenized for 1 min using a sonicator (Dr. Hielscher UP200s, Hielscher Ultrasonics GmbH, Teltow, Germany) and allowed to rest on ice for 1 min (repeated three times). The suspension was then centrifuged (10,000 rpm) for 20 min at 4 °C. The supernatants were filter-sterilized using a 0.22 µm surfactant-free cellulose acetate (SFCA) membrane filter (Sartorius Stedim Biotech, Goettingen, Germany) [29] and kept at −80 °C until use.

### 2.2. Cell Culture and Treatment

The SW1353 chondrosarcoma cell line, originally obtained from a human cell line, was procured from the Bioresource Collection and Research Center (BCRC) at the Food Industry Research and Development Institute in Hsinchu, Taiwan. The cells were maintained at 37 °C in Leibovitz’s L-15 medium (L-15) supplemented with 100 units/mL of penicillin, 100 mg/mL of streptomycin (Gibco BRL, Grand Island, NY, USA), and 10% fetal bovine serum (FBS) (HyClone, Auckland, NZ, USA) in a CO_2_-free incubator. The cells were seeded at the specified density onto 6 cm dishes or 24 well plates in L-15 medium and incubated for 16 h to allow for attachment. They were then treated with LDL557 extracts for 24 h, followed by treatment with or without MIA (Sigma-Aldrich Co., LLC, St. Louis, MO, USA) for an additional 24 h, after which various parameters were analyzed.

### 2.3. Cell Viability Assay

SW1353 cells were placed in each well of a 24 well plate at a density of 4 × 10^4^ cells per well. The cells were exposed to varying concentrations of LDL557 extracts, ranging from 0 to 200 μg/mL for 24 h, followed by incubation with or without 5 μM MIA for an additional 24 h. Once the treatments were completed, the cells were fixed with 95% alcohol and stained with 0.3% crystal violet (Sigma-Aldrich Co., LLC, St. Louis, MO, USA). The stain was then dissolved in a 0.1% acetic acid and 50% ethanol solution, and absorbance was measured at 595 nm. All experiments were performed in triplicate to ensure the accuracy of the results.

### 2.4. Toluidine Blue O Staining

Toluidine blue dyes can bind to anionic glycoconjugates (AGs) like proteoglycans (PGs) and glycosaminoglycans (GAGs), forming complexes [30]. SW1353 cells were seeded in 24 well plates at a density of 4 × 10^4^ cells per well and then treated with 10 μg/mL LDL557 extracts for 24 h, with or without adding 5 μM MIA for an additional 24 h. The cells were fixed with 4% paraformaldehyde (PFA) and stained with 0.5% toluidine blue O. After washing with PBS, images were captured using a digital camera (Olympus Corporation, Tokyo, Japan).

### 2.5. Real-Time Quantitative PCR Analysis (RT-qPCR)

The SW1353 cells were placed in a 6 cm dish at a density of 4 × 10^5^ cells per dish and then treated with 10 μg/mL LDL557 extracts for 24 h, with or without 5 μM MIA for an additional 24 h. After treatment, the total RNA was isolated from the cells using TRIzol™ reagent (Thermo Fisher Scientific Inc., Waltham, MA, USA), following the manufacturer’s protocol. Subsequently, cDNA synthesis was performed using 250 ng of random primers, 200 μM dNTPs, 200 units of recombinant RNasin ribonuclease inhibitor, and 200 units of M-MLV reverse transcriptase (Promega Corporation, Madison, WI, USA) with the extracted total RNA. Real-time quantitative PCR analysis (RT-qPCR) was performed on a CFX Duet Real-Time PCR System (Bio-Rad Laboratories, Hercules, CA, USA) using 100 ng of cDNA, 10 μL of Fast SYBR Green Master Mix (Applied Biosystems, Thermo Fisher Scientific, Waltham, MA, USA), and 200 nM of gene-specific primers for the target genes. Melting curve analysis confirmed the amplification specificity. Thermal cycling conditions included an initial denaturation at 95 °C for 10 min, followed by 40 cycles at 95 °C for 15 s and annealing at 60 °C for 60 s. After RT-qPCR, a melting curve was generated, with a temperature increase from 70 to 95 °C at a rate of 0.2 °C per second. The primer sequences and amplified products for each gene are listed in Table 1, with specificity verified using NCBI Primer-BLAST. The RT-qPCR procedures adhered to the MIQE guidelines [31]. The target gene Ct values were normalized to β-actin, and the results were analyzed and presented as 2^−ΔΔCt^ [32] using CFX Maestro Software 2.3 (Bio-Rad Laboratories, Hercules, CA, USA).

### 2.6. Oxidative Stress Analysis

The SW1353 cells were seeded in 6 cm dishes at a density of 4 × 10⁵ cells per dish and treated with 10 μg/mL LDL557 extracts for 24 h, followed by an additional 24 h of treatment with or without 5 μM MIA. Oxidative stress markers, including ROS, intracellular mitochondrial H_2_O_2_, and NO, were assessed using specific fluorescent probes: 2′,7′-dichlorodihydrofluorescein diacetate (DCFH-DA) for ROS (Molecular Probes, Eugene, OR, USA) [33], MitoPY1 for H_2_O_2_ (Tocris Bioscience, Bristol, UK) [34], and 4-amino-5-methylamino-2′,7′-difluorofluorescein diacetate (DAF-FM DA) for NO (Cayman Chemical Company, Ann Arbor, MI, USA) [35]. Measurements were performed following the manufacturer’s instructions with an Attune NxT AFC2 Acoustic Focusing Cytometer (Thermo Fisher Scientific, Waltham, MA, USA), and the data were analyzed with FCSalyzer version 0.9.22 (Sven Mostböck, Vienna, Austria). Each experiment was conducted in triplicate.

### 2.7. Statistical Analysis

Normality was assessed using the Shapiro–Wilk test, while the homogeneity of variance was checked using Levene’s test. The results indicated a normal distribution and had homogeneous variances. Subsequently, one-way ANOVA, followed by Tukey’s post hoc tests, was used to compare the groups, with statistical analyses performed using GraphPad Prism 8.1.1 (GraphPad Software, San Diego, CA, USA). A *p* value <0.05 was considered statistically significant. The 95% confidence intervals (CIs) are provided in Supplementary Figure S2.

## 3. Results

### 3.1. LDL557 Extracts Reduce MIA-Induced Cell Death

**Figure 1:**
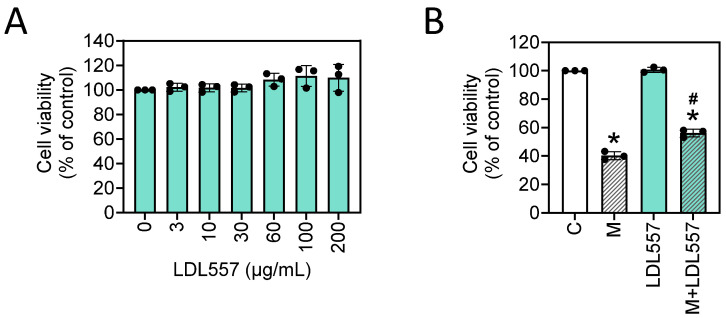
Effects of LDL557 extracts (LDL557) on SW1353 cell viability and monosodium iodoacetate (MIA, M)-induced cell death. The SW1353 cells were incubated for 24 h in the absence or presence of LDL557 extracts in the range of 3–200 μg/mL (**A**), or the cells were treated with 10 μg/mL LDL557 extracts for 24 h, followed by incubation with or without 5 μM MIA for an additional 24 h. Then, cell viability was determined via crystal violet staining (**B**). Data are presented as a percentage of the control group, accompanied by the mean ± standard deviation (S.D.) from three experiments. Statistically significant differences are indicated by * (*p* < 0.05 compared with the untreated control group) and # (*p* < 0.05 compared with the MIA-treated group). C = control untreated cells; M = MIA-treated cells; LDL557 = cells treated only with LDL557 extracts; M+LDL557 = cells treated with LDL557 extracts followed by treatment with MIA.

Our study found that the LDL557 extracts protected the SW1353 cells from MIA-induced damage. Initially, we tested the cytotoxicity of the LDL557 extracts in the SW1353 cells, and no cytotoxic effects were observed at concentrations ranging from 3 to 200 μg/mL (Figure 1A). We then challenged the cells with 5 μM MIA to mimic OA conditions, as demonstrated in our previous study [33], where MIA was used to induce similar cellular stress in chondrocytes. Cell viability assays revealed that the treatment with 1–10 μg/mL LDL557 extracts significantly improved cell viability compared with the MIA-only treatment group (Supplementary Figure S1). We selected the 10 μg/mL dose for subsequent experiments based on these results. No further increase in cell viability was observed at doses above 10 μg/mL, indicating that the protective effect plateaued at this concentration. As shown in Figure 1B, MIA reduced cell viability, but the addition of LDL557 extracts increased cell viability, indicating that the LDL557 extracts helped protect the chondrocytes from MIA-induced cytotoxicity.

### 3.2. LDL557 Extracts Inhibited MIA-Induced Extracellular Matrix Component Degradation

**Figure 2:**
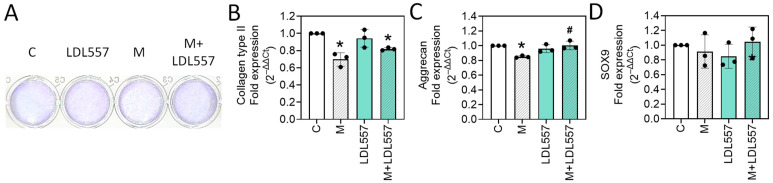
The effects of LDL557 extracts or MIA on extracellular matrix component synthesis. The details of the treatment with LDL557 extracts and MIA are described in Section 2.4 and Section 2.5. The acidic glycosaminoglycan contents were determined through toluidine blue O staining (**A**). The mRNA levels of collagen type II (**B**), aggrecan (**C**), and SOX9 (**D**) were analyzed using RT-qPCR, with β-actin as the internal control. Results are presented as fold changes relative to the untreated control. Data are presented as a percentage of the control group, accompanied by the mean ± standard deviation (S.D.) from three experiments. Statistically significant differences are indicated by * (*p* < 0.05 compared with the untreated control group) and # (*p* < 0.05 compared with the MIA-treated group). C = control untreated cells; M = MIA-treated cells; LDL557 = cells treated only with LDL557 extracts; M+LDL557 = cells treated with LDL557 extracts followed by treatment with MIA.

To investigate the effects of LDL557 extracts and MIA on extracellular matrix component synthesis, SW1353 cells were treated with LDL557 extracts with or without 5 μM MIA. Acidic polysaccharide levels were examined using toluidine blue O staining, and the gene expressions of collagen type II and aggrecan, as well as their upstream regulator of transcription factor SOX9, were analyzed with RT-qPCR. The results showed that the MIA challenge significantly decreased the levels of glycosaminoglycans (Figure 2A) and the expression of collagen type II (Figure 2B) and aggrecan (Figure 2C), whereas the expression of the transcription factor SOX9 was unaffected (Figure 2D). Aside from this, adding LDL557 extracts could significantly lessen the decrease in matrix contents and the expression of aggrecan. In contrast, SOX9 expression was not affected.

The primary outcomes of this study were evaluated to assess the therapeutic potential of LDL557 extracts in protecting chondrocyte viability and integrity under OA-mimicking conditions. First, “protection against cell death” was evaluated by measuring cell viability in the SW1353 cells exposed to MIA. The results showed that MIA treatment significantly reduced cell viability, whereas adding LDL557 extracts (10 μg/mL) restored cell viability, indicating a protective effect against MIA-induced cytotoxicity. Second, “reduction in ECM loss” was evaluated via toluidine blue O staining, which demonstrated changes in glycosaminoglycan levels. MIA exposure significantly decreased the GAG content, but treatment with LDL557 extracts mitigated this reduction, highlighting the extracts’ potential to protect against ECM degradation. These findings suggest that LDL557 extracts protect chondrocytes from MIA-induced damage and help preserve cartilage integrity in an in vitro OA model.

### 3.3. LDL557 Extracts Did Not Change MIA-Induced Matrix Metalloproteinase (MMP) mRNA Expression Levels

**Figure 3:**
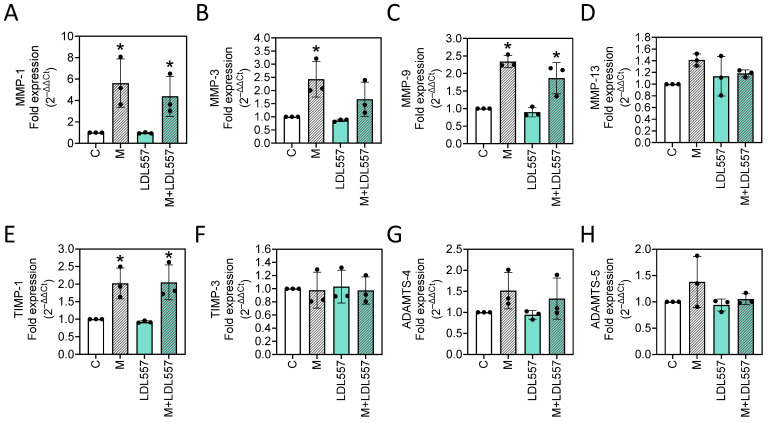
The effects of LDL557 extracts or MIA on matrix metalloproteinase (MMP) mRNA expression. The details of the treatment with LDL557 extracts and MIA are described in Section 2.5. The expressions of MMP-1 (**A**), MMP-3 (**B**), MMP-9 (**C**), MMP-13 (**D**), TIMP-1 (**E**), TIMP-3 (**F**), ADAMTS-4 (**G**), and ADAMTS-5 (**H**) were assayed via RT-qPCR with β-actin as the internal control. Results are presented as fold changes relative to the untreated control. Data are presented as the fold change of the control group accompanied by the mean ± standard deviation (S.D.) from three experiments. Statistically significant differences are indicated by * (*p* < 0.05 compared with the untreated control group). C = control untreated cells; M = MIA-treated cells; LDL557 = cells treated only with LDL557 extracts; M+LDL557 = cells treated with LDL557 extracts followed by treatment with MIA.

To investigate whether LDL557 extracts or MIA affect MMPs, including MMP-1, MMP-3, MMP-9, and MMP-13, or aggrecanases, such as ADAMTS-4 and ADAMTS-5, the mRNA expressions were analyzed using RT-qPCR. Meanwhile, the expressions of the MMP regulators, including TIMP-1 and TIMP-3, were also assayed. The results show that MIA significantly increased the expression of MMP-1 (Figure 3A), MMP-3 (Figure 3B), MMP-9 (Figure 3C), and TIMP-1 (Figure 3E) and the upward trend of MMP-13 expression (C versus M, *p* = 0.0851, Figure 3D). However, among the MMPs, the addition of LDL557 extracts only slightly reduced MMP-3 expression and did not reach statistical significance (Figure 3B). Furthermore, the LDL557 extracts did not affect the aggrecanases or regulators of MMPs (Figure 3E–H). This indicates that LDL557 extracts do not affect TIMPs to then affect MMP or ADAMTS expression.

### 3.4. LDL557 Extracts Did Not Change Oxidative Stress

**Figure 4:**
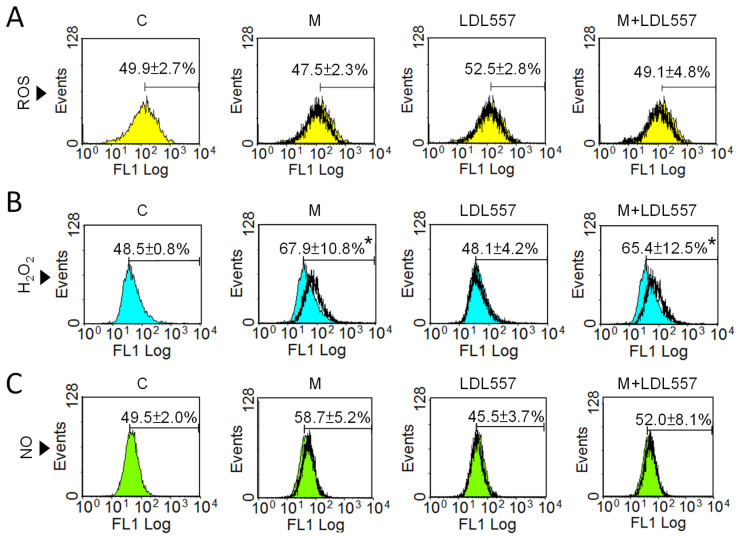
Oxidative stress of SW1353 cells treated with LDL557 extracts or MIA. The details of the treatment with LDL557 extracts and MIA are described in Section 2.6, and flow cytometry was conducted to analyze the levels of ROS (**A**), H_2_O_2_ (**B**), and NO (**C**) using DCFH-DA, MitoPY1, and DAF-FM DA staining, respectively. The untreated control is depicted by the color-filled area (yellow, blue, or green), while the treated groups are represented by the thick black lines. Data are presented as a percentage of the control group, accompanied by the mean ± standard deviation (S.D.) from three experiments. Statistically significant differences are indicated by * (*p* < 0.05 compared with the untreated control group). C = control untreated cells; M = MIA-treated cells; LDL557 = cells treated only with LDL557 extracts; M+LDL557 = cells treated with LDL557 extracts followed by treatment with MIA.

It has been reported that oxidative stress is increased in OA [36]. Accordingly, we investigated whether the LDL557 extracts effectively reduced oxidative stress in the SW1353 cells. The ROS (Figure 4A), H_2_O_2_ (Figure 4B), and NO (Figure 4C) levels were analyzed via flow cytometry. The results show that MIA had no impact on the levels of ROS and NO, but it did increase the H_2_O_2_ levels (Figure 4B). In addition, the LDL557 extracts did not exhibit any effect either with the MIA challenge or not, suggesting that the effects of the LDL557 extracts were unrelated to the change in oxidative stress.

### 3.5. LDL557 Extracts Decreased the MIA-Induced Inflammatory Cytokine mRNA Expression

**Figure 5:**
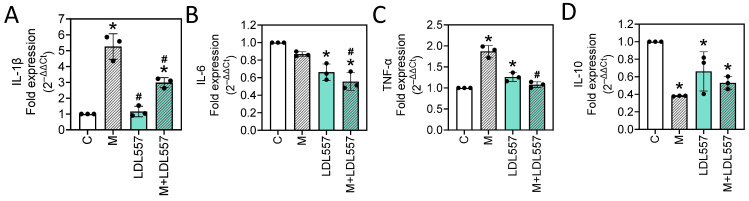
Effects of LDL557 extracts or MIA on inflammatory and anti-inflammatory cytokines’ mRNA expression in SW1353 cells. The details of the treatment with LDL557 extracts and MIA are described in Section 2.5. The mRNA expression levels of IL-1β (**A**), IL-6 (**B**), TNF-α (**C**), and IL-10 (**D**) were analyzed via RT-qPCR with β-actin used as the internal control. Data are presented as a fold change of the control group, accompanied by the mean ± standard deviation (S.D.) from three experiments. Statistically significant differences are indicated by * (*p* < 0.05 compared with the untreated control group) and # (*p* < 0.05 compared with the MIA-treated group). C = control untreated cells; M = MIA-treated cells; LDL557 = cells treated only with LDL557 extracts; M+LDL557 = cells treated with LDL557 extracts followed by treatment with MIA.

To determine whether the inflammatory cytokines of IL-1β, IL-6, and TNF-α and the anti-inflammatory cytokines IL-10 were involved in the effects of the LDL557 extracts or MIA on SW1353 cells, the expression levels of IL-1β, IL-6, TNF-α, and IL-10 were analyzed using RT-qPCR. As shown in Figure 5, the mRNA expressions of inflammatory cytokines IL-1β (Figure 5A) and TNF-α (Figure 5C) significantly increased following MIA treatment, while that of anti-inflammatory cytokine IL-10 (Figure 5D) was reduced. Notably, the addition of LDL557 extracts mitigated the MIA-induced increases in IL-1β and TNF-α expression and showed a trend toward increasing IL-10 expression (M versus M+LDL557, *p* = 0.06914, Figure 5D). These findings suggest that LDL557 extracts exert direct regulatory effects on the expression of MIA-induced cytokines.

### 3.6. LDL557 Extracts May Decrease Cyclooxygenase-2 (COX-2) mRNA Expression

**Figure 6:**
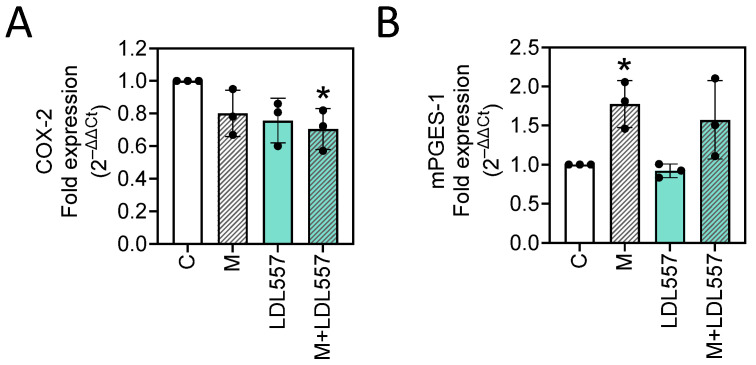
Effects of LDL557 extracts or MIA on COX-2 and mPGES-1 mRNA expression in SW1353 cells. The details of the treatment with LDL557 extracts and MIA are described in Section 2.5. The mRNA expressions of COX-2 (**A**) and mPGES-1 (**B**) were measured with β-actin used as the internal control. Data are presented as a fold change of the control group, accompanied by the mean ± standard deviation (S.D.) from three experiments. Statistically significant differences are indicated by * (*p* < 0.05 compared with the untreated control group). C = control untreated cells; M = MIA-treated cells; LDL557 = cells treated only with LDL557 extracts; M+LDL557 = cells treated with LDL557 extracts followed by treatment with MIA.

To investigate the roles of COX-2 and mPGES-1 in the effects of LDL557 extracts or MIA on SW1353 cells, we quantified their gene expressions using RT-qPCR. The results show that MIA treatment significantly increased mPGES-1 expression (Figure 6B) but did not alter COX-2 expression (Figure 6A). Interestingly, the addition of LDL557 extracts showed a trend of reduced COX-2 expression (C versus LDL557, *p* = 0.0530), and a statistically significant reduction was observed in the MIA-treated group (C versus M+LDL557, *p* < 0.05). Meanwhile, mPGES-1 expression remained unaffected by the LDL557 extracts in the MIA-treated group compared with the MIA-only group. This indicates that LDL557 extracts may exert biological effects by decreasing COX-2 expression, thereby reducing inflammation.

## 4. Discussion

In this study, LDL557 extracts demonstrated protective effects against MIA-induced cell death and significantly reduced key cytokines, including the mRNA expression levels of IL-1β, IL-6, and TNF-α (Figure 5), resulting in increased GAG synthesis (Figure 2A). These findings highlight their potential in mitigating inflammation associated with OA. Furthermore, LDL557 extracts slightly increased the mRNA expression of IL-10, an anti-inflammatory cytokine with known chondroprotective effects, in MIA-treated cells. Although LDL557 extracts did not significantly influence oxidative stress markers such as the H_2_O_2_, ROS, or NO levels, their ability to reduce pro-inflammatory cytokines suggests a targeted mechanism focused on cytokine modulation rather than oxidative stress pathways.

In cartilage, pro-inflammatory cytokines such as IL-1β and TNF-α have been shown to upregulate the expression of both COX-2 and mPGES-1, contributing to the inflammatory process and cartilage degradation in patients with OA [37]. In this study, we found that the LDL557 extracts significantly reduced the mRNA expression levels of key pro-inflammatory cytokines such as IL-1β, IL-6, and TNF-α, highlighting their potential role in attenuating the cartilage degradation commonly associated with OA. These findings suggest that the primary anti-inflammatory action of LDL557 extracts may stem from their ability to modulate cytokine production rather than suppressing the COX-2/mPGES-1 pathway directly. However, the extracts did not provide significant protection against MIA-induced mRNA expression of mPGES-1 upregulation, indicating a more selective mechanism of action. Interestingly, while the LDL557 extracts demonstrated a reduction in COX-2 mRNA expression compared with the control group, their protective effects appeared to be diminished under the harmful conditions induced by MIA. Moreover, the lack of a significant increase in COX-2 mRNA expression following MIA treatment alone suggests that the observed reduction in COX-2 by the LDL557 extracts was independent of MIA-induced alterations. To build on these findings, further investigations are needed to unravel the interactions between LDL557 extracts and key inflammatory pathways, particularly through time-optimized experiments and validation of effects at the protein level.

Currently, there are various anti-inflammatory adjuvant therapies available to improve OA. These include biological agents, nonsteroidal anti-inflammatory drugs (NSAIDs), and corticosteroids. For instance, adalimumab, a monoclonal antibody targeting TNF-α, has been shown to effectively relieve pain in patients with severe OA. Additionally, systemic blockading of IL-6 using an anti-IL-6 receptor-neutralizing antibody has been found to reduce cartilage lesions, osteophyte formation, and the severity of synovitis in mice with DMM-induced OA [38]. Despite these advancements, existing therapies like oral and topical NSAIDs and intra-articular corticosteroid injections come with limitations. Prolonged use of NSAIDs can result in systemic side effects, while repeated corticosteroid injections may lead to potential damage to cartilage [39].

Probiotics have emerged as a complementary approach which can improve gut health and modulate immune responses by suppressing pro-inflammatory cytokines and stimulating anti-inflammatory cytokines [40]. Among these, *Lactobacillus delbrueckii*, a key species in the gastrointestinal tract (GIT), has been extensively studied for its probiotic potential, including resistance to GIT stressors, pathogen inhibition, and anti-inflammatory effects. These benefits have primarily been explored in GIT-related conditions such as colorectal cancer, ulcerative colitis, and intestinal mucositis [41].

Postbiotics, derived from inanimate probiotics, are gaining recognition as viable alternatives due to their enhanced stability, safety, and efficacy. It has been shown that postbiotics from *Lactobacillus delbrueckii* subsp. *jakobsenii* significantly decreased IL-6 and TNF-α expression levels while increasing IL-10 levels in the serum and jejunum of *Salmonella typhimurium*-infected mice, indicating their ability to influence inflammatory factor secretion [42], which is in accordance with our findings. Similarly, a study on LDL557 by Chen et al. revealed that both live and heat-killed LDL557 significantly reduced methacholine-induced airway hyper-responsiveness, inflammation, and mucus production, with the heat-killed LDL557 demonstrating a more substantial capacity to alter the gut microbiota composition [43]. These findings contribute to the growing body of evidence supporting the role of postbiotics in mitigating disease conditions.

Notably, our previous findings showed that the heat-killed LDL557 exhibited protective effects which were equivalent to or even more pronounced than those of live LDL557 in the MIA-induced OA rat model [27]. This approach provided a cellular-level exploration of the therapeutic potential of LDL557 extracts, in which LDL557 extracts at 10 μg/mL (4.3 × 10^6^ cells/mL) were identified as the optimal dose, since higher concentrations did not more effectively protect against MIA-induced cell death (Supplementary Figure S1). Importantly, these findings suggest that LDL557 extracts could be developed into oral supplements with the potential to mitigate OA progression significantly. This conclusion is supported by our prior in vivo studies using heat-killed LDL557 (5.14 × 10⁹ cells/kg body weight in rats) [27]. The doses were calculated based on a human equivalent dose (HED) derived from body surface area conversion, estimating a daily human dose of approximately 5 × 10¹⁰ cells/day [44].

Heat-killed LDL557 offers distinct practical advantages, including stability and an extended shelf life, making it well suited for clinical and commercial applications. Moreover, the absence of adverse effects in animal studies supports the safety of this approach. Genomic analysis further supports the safety profile of LDL557. The whole-genome sequence analysis revealed no antimicrobial resistance or virulence-associated genes, and protein family identification confirmed its non-pathogenic nature. As a strain of *Lactobacillus delbrueckii*, LDL557 is proposed for QPS status due to its established history of safe use [28].

Although postbiotics are increasingly recognized for their health benefits, their application in OA management has been relatively underexplored. This study introduces the concept of employing postbiotics, specifically LDL557 extracts, as a potential therapeutic approach for OA. However, certain limitations exist, including using a cell line rather than primary cells, unclear identification of the specific active components in LDL557 extracts, and the absence of protein-level data. While our findings demonstrated anti-inflammatory and chondroprotective effects in vitro, clinical validation is necessary. Future studies should evaluate long-term effects in human trials, optimize dosing strategies, and investigate systemic impacts, including modulation of gut microbiota and inflammation.

This study indicates that LDL557 extracts support joint health by reducing inflammation-related factors, thereby mitigating cartilage matrix loss and promoting joint protection. While the precise health-promoting mechanisms of LDL557 extracts remain unclear, their beneficial effects are partially mediated through immunomodulation, contributing to multiple health benefits. As food supplements, postbiotics offer practical advantages over probiotics, including enhanced stability, easier storage, and an extended shelf life, making them an appealing alternative for health-focused food applications.

## 5. Conclusions

This study aimed to explore the effectiveness of LDL557 extracts on chondrocytes in OA. The experimental results showed that LDL557 extracts may protect chondrocytes from OA damage by reducing the inflammation-related factors of pro-inflammatory cytokines, thus mitigating cartilage matrix loss. This suggests that LDL557 extracts are an attractive alternative for OA applications. Overall, this study confirms the efficacy of LDL557 extracts on chondrocytes in the OA status, inferring LDL557 extracts have the potential to be attractive alternatives for joint health.

## Figures and Tables

**Table 1:** Primer sets for qPCR analysis.

Primer Name	NCBI Reference Sequence	Primer Sequence (5′→3′)	Amplicon Length (bp)
β-actin	NM_001101.3	F: ATCGGCGGCTCCATCCTG R: ACTCGTCATACTCCTGCTTGC	73
Collagen type II	NM_001844.4	F: GCAGCAAGAGCAAGGAGAAG R: GTGGACAGCAGGCGTAGG	136
Aggrecan	NM_001135.3	F: GCAGCAAGAGCAAGGAGAAG R: GTGGACAGCAGGCGTAGG	128
SOX9	Z46629.1	F: TCTGAACGAGAGCGAGAAGC R: CCGTTCTTCACCGACTTCCT	120
MMP-1	NM_002421.3	F: AGATGTGGAGTGCCTGATGTG R: CTTGACCCTCAGAGACCTTGG	199
MMP-3	NM_002422.3	F: CCACTCTATCACTCACTCACAG R: GACAGCATCAAAGGACAAAGC	185
MMP-9	NM_004994.2	F: CTGGTCCTGGTGCTCCTG R: TGCCTGTCGGTGAGATTGG	110
MMP-13	NM_002427.3	F: GACCCTGGAGCACTCATGTTTC R: TCCTCGGAGACTGGTAATGGC	192
ADAMTS-4	NM_005099.4	F: AGGCAGTGATGGGTTAGTGG R: CCTAGTCCTTGTCCCCTTCC	178
ADAMTS-5	NM_007038.3	F: GCTACTGCACAGGGAAGAGG R: GGCAGGACACCTGCATATTT	172
TIMP-1	NM_003254	F: GCGGATACTTCCACAGGTCC R: GCTAAGCTCAGGCTGTTCCA	124
TIMP-3	NM_000362	F: GGAAGAGAGTACCGGCATCG R: CTTCTGCTCACACTGCCTCA	132
IL-1β	NM_000576.2	F: TGATGGCTTATTACAGTGGCAATG R: GTAGTGGTGGTCGGAGATTCG	140
IL-6	NM_000600.4	F: ACCCCCAATAAATATAGGACTGGA R: GAGAAGGCAACTGGACCGAA	145
TNF-α	NM_000594.3	F: TCAGCAAGGACAGCAGAGGAC R: GGAGCCGTGGGTCAGTATGTG	138
IL-10	NM_000572.2	F: GGCTTCCTAACTGCTACAAATAC R: AATCCCTCCGAGACACTGG	89
COX-2	NM_000963.3	F: AAGTCCCTGAGCATCTACGGTTTG R: TGTTCCCGCAGCCAGATTGTG	94
mPGES-1	NM_004878.5	F: CCCTGAGTCCTGGTTTCCTG R: GTTCCCATCAGCCACTTCGT	142

MMP = matrix metallopeptidase; ADAMTSs = a disintegrin and metalloproteinase with thrombospondin motifs; TIMPs = tissue inhibitors of metalloproteinases; IL = interleukin; TNF = tumor necrosis factor; COX-2 = cyclooxygenase-2; mPGES-1 = microsomal prostaglandin E synthase-1; F = forward primer; R = reverse primer.

## Data Availability

Data are contained within this article and the Supplementary Materials.

## Supplementary Materials

The following supporting information can be downloaded at https://www.mdpi.com/article/10.3390/nu16244417/s1. Figure S1: The cell viability of cells treated with 0–100 μg/mL LDL557 extracts in response to 5 μM MIA-induced cell death. Figure S2: The actual numerical information of the 95% CI between each group.
