# Supplementary material for: Heat-Killed Lactobacillus delbrueckii subsp. lactis 557 Extracts Protect Chondrocytes from Osteoarthritis Damage by Reducing Inflammation: An In Vitro Study
Source: Nutrients. 2024 Dec 23;16(24):4417. doi: 10.3390/nu16244417 (PMC11676954; doi:10.3390/nu16244417)
Supplement: Supplementary file 1 [file nutrients-16-04417-s001.zip › nutrients-3351561-supplementary.pdf]

Supplementary Figure S1

The cell viability of cells treated with 0–100 μg/mL LDL557 extracts in response to 5 μM MIA-induced cell death is shown in the table. The data is expressed as a percentage of the control and represented as the mean ± S.D. for three independent experiments. Statistically significant differences are indicated by * ($p < 0.05$ compared to the untreated control group) and # ($p < 0.05$ compared to the MIA-only treated group).

		LDL557 extracts (μg/mL)					
		0	1	3	10	30	100
MIA (μM)	0	100±0.5	101.6±1.6	102.5±0.6	100.6±1.8	105.7±2.3	109.2±3.9
	5	40.3±2.8*	46.9±1.5*#	50.4±1.4*#	56.2±2.7*#	43.5±1.0*	36.8±2.0*

Supplementary Figure S2(A)-(B)
The actual numerical information of the 95% CI between each group is shown in the table.

(A)

LDL557 extracts (μg/mL)	95% CI (Figure 1A)
0	100–100
3	94.3–110.2
10	93.7–109.8
30	94.0–109.3
60	95.4–121.4
100	90.4–132.2
200	82.9–136.9

(B)

	95% CI			
	C	M	LDL557	M+LDL557
Cell viability (Figure 1B)	100–100	33.3–47.2	96.2–105.1	49.5–62.9
Collagen type II	1.00–1.00	0.50–0.70	0.69–0.94	0.78–0.82
Aggrecan	1.00–1.00	0.81–0.89	0.83–1.09	0.86–1.15
SOX9	1.00–1.00	0.55–1.28	0.63–1.06	0.73–1.35
MMP-1	1.00–1.00	2.11–9.17	0.89–1.04	1.5–7.27
MMP-3	1.00–1.00	1.46–3.27	0.81–0.93	0.81–2.49
MMP-9	1.00–1.00	2.06–2.58	0.66–1.13	1.08–2.64
MMP-13	1.00–1.00	1.16–1.67	0.30–1.97	1.04–1.33
TIMP-1	1.00–1.00	1.34–2.72	0.88–0.97	1.22–2.80
TIMP-3	1.00–1.00	0.54–1.41	0.62–1.45	0.64–1.30
ADAMTS-4	1.00–1.00	0.44–2.59	0.69–1.19	0.11–2.54
ADAMTS-5	1.00–1.00	0.17–2.58	0.65–1.22	0.79–1.31
ROS	43.2–56.6	41.9–53.1	45.6–59.3	37.2–60.9
H_2O_2	46.5–50.5	51.9–77.2	37.6–58.6	44.8–79.3
NO	44.7–54.4	45.9–71.5	36.3–54.6	31.9–72.2
IL-1β	1.00–1.00	3.27–7.26	0.33–1.98	2.20–3.77
IL-6	1.00–1.00	0.80–0.93	0.43–0.90	0.31–0.80
TNF-α	1.00–1.00	1.53–2.21	1.01–1.51	0.91–1.25
IL-10	1.00–1.00	0.37–0.39	0.11–1.22	0.35–0.71
COX-2	1.00–1.00	0.56–1.05	0.56–0.95	0.50–0.91
mPGES-1	1.00–1.00	1.25–2.30	0.79–1.06	0.70–2.45
